# Ultrasound-Assisted Extraction of Phenolic Compounds from *Psidium cattleianum* Leaves: Optimization Using the Response Surface Methodology

**DOI:** 10.3390/molecules27113557

**Published:** 2022-05-31

**Authors:** Napoleón González-Silva, Yolanda Nolasco-González, Gabriela Aguilar-Hernández, Sonia Guadalupe Sáyago-Ayerdi, Zuamí Villagrán, José Luis Acosta, Efigenia Montalvo-González, Luis Miguel Anaya-Esparza

**Affiliations:** 1 Department of Livestock and Agricultural Sciences, University Center of Los Altos, University of Guadalajara, Av. Rafael Casillas Aceves 1200, Guadalajara 47600, Mexico; napoleon.gonzalez@cualtos.udg.mx (N.G.-S.); gaby.mca2017@gmail.com (G.A.-H.); 2 Integral Food Research Laboratory, National Technological of Mexico/Technological Institute of Tepic, Av. Tecnologico 2595, Tepic 63175, Mexico; nolasco.yolanda@inifap.gob.mx (Y.N.-G.); ssayago@ittepic.edu.mx (S.G.S.-A.); 3 Department of Health Sciences, Division of Biomedical Science, University Center of Los Altos, University of Guadalajara, Av. Rafael Casillas Aceves 1200, Guadalajara 47600, Mexico; blanca.villagran@academicos.udg.mx; 4 Interdisciplinary Research Centre for Integral Regional Development Sinaloa Unit, National Polytechnic Institute, Boulevard Juan de Dios Bátiz 250, Guasave 81049, Mexico; jlacostar@ipn.mx

**Keywords:** *Psidium cattleianum* leaves, ultrasound-assisted extraction, optimization, polyphenols, antioxidant activity

## Abstract

In this study, conditions for the ultrasound-assisted extraction (UAE) of soluble polyphenols from *Psidium cattleianum* (PC) leaves were optimized using response surface methodology (RSM) by assessing the effect of extraction time (X_ET_ = 2, 4, and 6 min), sonication amplitude (X_SA_ = 60, 80, and 100%), and pulse cycle (X_PC_ = 0.4, 0.7, and 1 s). Furthermore, the optimized UAE conditions were compared with a conventional aqueous–organic extraction (AOE) method for extracting total phenolics; moreover, a phenolic profile using HPLC and antioxidant activity (DPPH, ABTS, and FRAP) were also compared. According to the RSM, the best conditions for UAE to extract the highest soluble polyphenol content and yield (158.18 mg/g dry matter [DM] and 15.81%) include a 100% sonication amplitude for 4 min at 0.6 s of pulse cycle. The optimal UAE conditions exhibited an effectiveness of 1.71 times in comparison to the AOE method for extracting total phenolics, in 96.66% less time; moreover, PC leaf extracts by UAE showed higher antioxidant values than AOE. Additionally, gallic, protocateic, chlorogenic, caffeic, coumaric, trans-cinnamic, 4-hydroxybenzoic, and syringic acids, as well as kaempferol were identified in PC leaves under UAE. PC leaf extracts are widely used for therapeutic and other industrial purposes; thus, the UAE proves to be a useful technology with which to improve the yield extraction of PC leaf phytochemicals.

## 1. Introduction

*Psidium cattleianum* (PC) Sabine is a member of the Myrtaceae family, and it is closely related to *Psidium guajava*, the most representative and commercially cultivated fruit of the *Psidium* genera [1]. PC is native to Brazil and is widely distributed in tropical and subtropical climate regions worldwide [1]. It is a shrub or small tree, which can reach 2.5 m in height, known as araçá or strawberry-guava that produces a small, yellow, or reddish fruit that is globose in shape [2]. PC leaves have a bright green color and exhibit relevant ethnobotanical importance [3]. In folk medicine practice, they have been used as anti-hemorrhagic, anti-diarrheal, and antispasmodic agents, as well as to relieve toothache and abdominal pain by chewing them or by brewing tea by infusion (dry leaves) or decoction (fresh leaves) [2,4]. Moreover, PC leaves demonstrated in vitro antimicrobial, hypoglycemic, and antitumoral properties [2,4,5]. Valentin et al. [5] reported that PC leaf ethanolic extracts exhibited favorable tissue repair, associated with their anti-inflammatory properties. Furthermore, Antonelli et al. [6] used the PC leaf extract as a pre-emergent bioherbicide in *Lactuca sativa* seeds. Recently, PC leaf extracts were integrated into the diet of laying hens due to their antioxidant and antimicrobial properties for the improvement of egg quality [7]. These effects were attributed to the content of polyphenols and the antioxidant activity of PC leaf extracts [4]. Therefore, PC leaves could be recognized as a source of bioactive compounds with antioxidant activity for potential industrial and pharmaceutical applications [4].

Phytochemicals or bioactive compounds are substances formed by the secondary metabolism of plants, where polyphenols are highlighted as the most extensive group [8]. In plants, these compounds play important physiological functions as a defense mechanism against plagues and predators; while simultaneously exhibiting potential health benefits in the human body due to their biological properties (i.e., anti-inflammatory, antimicrobial, and cardioprotective properties) [8,9]. Phenolic compounds are composed of one (or more) aromatic rings, with one (or more) hydroxyl groups that exhibit strong antioxidant activity by donating a hydrogen atom or an electron to free radicals [8]. In general, diverse bioactive compounds (saponins, steroids, alkaloids, tannins, triterpenoids, and essential oils) have been reported derived from PC leaf ethanolic extracts [3,10,11], including polyphenols such as gallic, ellagic, chlorogenic, vanillic, and syringic acids, as well as catechin, quercetin, kaempferol, and luteolin [4,12].

Traditionally, the extraction of polyphenols from PC leaves was performed using conventional methods (stirring, pressing, maceration, and hydrodistillation) with or without thermal treatments [13,14]. Different authors used decoction (100 g of dried leaves/600 mL deionized water at 100 °C for 5 min) [15,16,17], Soxhlet or heat reflux extraction (2 g of dried leaves/100 mL of hexane at 90 °C for 90 min) [18], hydro-distillation (for 2 to 5 h in a modified Clevenger apparatus) [19,20], stirring at room temperature (1.2 kg of dried leaves/12 L of 80% methanol for 3 days) [21,22], hand-shaking (20 g of dried leaves/250 mL of 80% ethanol, shaking vigorously 5 times a day for 12 days) [23], and maceration at room temperature with methanol (1.2 kg of dried leaves/12 L of 80% methanol for 14 days) [24] or ethanol (0.5 kg of dried leaves/500 mL for 5 days) [11] to extract bioactive compounds from PC leaves. However, these methods required high amounts of solvents and samples, and long extraction times that may affect the stability (oxidation, ionization, and hydrolysis), yield, and bioactivity of these phytochemicals [13,14]. On the other hand, alternative non-conventional extraction techniques such as enzymatic (using a cellulase complex) [12], liquid-pressurized [4], supercritical fluid [18], and ultrasound-assisted extraction [12,18] have been applied to extract bioactive compounds from PC leaves.

Ultrasound-assisted extraction (UAE) is labeled as a green extraction process with higher bioactive compound yields than conventional extraction methods and some non-conventional extraction methods [18]. UAE is a simple operation, inexpensive, fast, and efficient alternative to extracting bioactive compounds from plant materials [13]. The UAE process is based on the use of sound waves beyond human hearing (ultrasound in a range of 20 to 100 kHz) that pass through a liquid medium, producing cavitation (formation and collapse/implosion of bubbles) that significantly enhances the mass transfer rates in the process with high solvent penetration power into cell materials, promoting changes in the plant material by its physical and mechanical effects, facilitating the extraction of bioactive compounds from the plant matrix [9,14,25]. In recent years, UAE has been investigated for the extraction of polyphenols from diverse plant leaf materials, including starfruit [26], muicle [27], jackfruit [28], olive [29], and moringa [30]. Similarly, UAE has been employed to extract bioactive compounds from *Psidium cattleianum* fruit peel [9] and leaves [12,18]. In PC leaves, indirect and direct UAE processes were investigated using an ultrasound water bath [12] and an ultrasonic sonicator probe [18] to extract polyphenols.

According to Zandoná et al. [4], optimizing the extraction conditions increases the amount of released bioactive compounds from plant material, particularly when UAE is applied [9]. Therefore, in the UAE, it is necessary to evaluate different factors that influence the extraction process, such as frequency, sound wave amplitude, pulse cycle, acoustic power, temperature, and extraction time, among others [13,14]. The Box–Behnken design and response surface methodology (RSM) are statistical and mathematical strategies used to optimize extraction procedures, providing large amounts of information with a small number of experiments (economic approach), which is based on the evaluation of the relationship (effects and interactions) between extraction factors and the response variable [25,26,27] that significantly describes the extraction process [9]. Recently, it has been reported that leaf/solvent ratio, temperature, and acoustic power have a significant effect on the extraction of phenolic compounds from PC leaves by the UAE [18].

This work aimed to optimize the UAE conditions (pulse cycle, extraction time, and sonication amplitude) on the content of soluble polyphenols from *Pisidium cattleainum* leaves using response surface methodology. Additionally, the optimized UAE conditions were compared with a conventional aqueous–organic extraction method to extract polyphenolic compounds and identify the antioxidant capacity, yield, effectiveness, and phenolic profile using HPLC.

## 2. Results and Discussion

**Figure 1:**
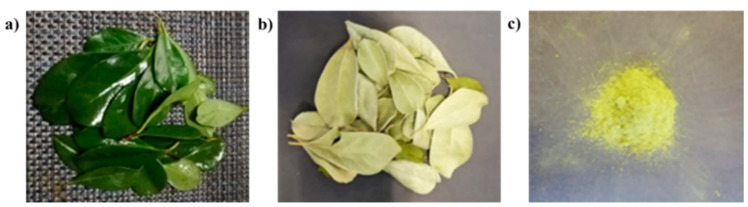
Fresh (**a**), dried (**b**), and powder leaves (**c**) of *Psidium cattleianum*.

*Psidium cattleianum* leaves were selected according to a uniform green coloration, excluding material with rottenness, defects, or injuries (Figure 1), as recommended by Dacoreggio et al. [12].

### 2.1. Ultrasound-Assisted Extraction (UAE) of Soluble Polyphenols from Psidium cattleianum Leaves

The Box–Behnken experimental design, experimental data, and yield for soluble polyphenols obtained from PC leaf extracts by UAE are listed in Table 1. Significant differences (*p* < 0.05) were observed between treatments, where the phenolic extraction yield (from 11.62 to 15.95%) was handled in an experimental conditions-dependent manner. Coelho et al. [18] reported a phenolic extraction yield of 2.55% after applying direct UAE (20 kHz, 5 min) in PC leaves when evaluating the temperature (40, 50, and 60 °C), power ultrasound (100, 300, and 500 W), and leaf-solvent ration (1:10, 1:15, and 1:20), using a full factorial design 2^3^. The maximum UAE of the total soluble phenols (159.55 mg/g DM) from the PC leaf powder was found at experimental conditions of a pulse cycle of 0.7 s, sonication amplitude of 60%, and extraction time of 2 min. On the other hand, the lowest soluble phenols content (116.24 mg/g DM) was observed at a low pulse cycle (0.4 s) and extraction time of 2 min, and 80% sonication amplitude. Furthermore, these values were higher than those reported by Dacoreggio et al. [12], who reported a total phenolic content of 101 mg/g from PC leaves by UAE, using an ultrasound bath (indirect ultrasound) at 70 W for 90 min. Rohilla et al. [25] highlighted that the sonication amplitude plays a critical role in the AUE of bioactive compounds from plant materials. They described there being a decrease in soluble phenols from *Solanum betaceum* fruit when increasing the sonication amplitude from 50 to 70% and explained that the degradation of phenolic compounds due to an increase in amplitude beyond optimum level occurs. In this study, the time required to obtain a higher soluble phenol content from PC leaf extract was reduced by 98% [12], which is important as longer extraction times can cause phenolic degradation, particularly in PC leaf extract [18]. This comparison shows the importance of optimizing UAE extraction conditions. The increase in the soluble phenolic content is associated with direct contact between the ultrasonic probe and leaf extract, enhancing the physical and mechanical effects of the cavitation phenomenon that destroys the cell-wall structure and produces a porous surface, facilitating the release of phenolic compounds [27]. Additionally, during the UAE process, the temperature was kept at 20 ± 1 °C, and it has been reported that the UAE of phenols from PC leaves at low temperatures can help to avoid polyphenol degradation and an increase the extraction yield [18]. A considerable number of studies have demonstrated that UAE (ultrasound coupled with a sonicator probe) could be used as a technological, viable, and efficient method for extracting phenolic compounds from plant-based materials, including fruit peel, seeds, and leaves, among others [25,26,27,31,32].

### 2.2. Response Surface Methodology Analysis

A response surface methodology (RSM) analysis was performed to obtain the optimal UAE conditions for extracting phenolic compounds from PC leaves. Statistically significant effects of the independent factors (X_PC_, X_SA_, and X_ET_) on the UAE of the total soluble phenols were verified by the analysis of variance (ANOVA, *p* < 0.05) and regression coefficients of the experimental model using multiple regression analysis (Table 2). The ANOVA showed that most of the factors and their interactions (except for X_ET_^2^ and X_PC_) were significant (*p* < 0.05); moreover, they exhibited a significant correlation coefficient (R^2^ = 0.9925) and adequate experimental data for the estimated model (Lack of fit, *p* > 0.05), suggesting an approximation to a real system [31,33]. Various authors have reported correlation coefficients ranging from 0.85 to 0.99 when applying UAE to extract bioactive compounds from plant materials (pomegranate and strawberry-guava peel, soursop peel, seeds, columella, and pulp, and starfruit and muicle leaves) using full factorial, central composite rotational, and Box–Behnken designs and an RSM analysis [9,26,27,34,35], demonstrating that RSM is an efficient statistical tool to optimize UAE conditions in obtaining phenolic compounds from plant-based materials, particularly for PC leaves.

Additionally, all β-coefficients of the fitted regression model (Table 2) were significant (*p* < 0.05). According to the polynomial equation (second-order), the total soluble phenols content from PC leaf extracts using UAE can be predicted (R^2^ = 0.9925, R^2^ adj. = 0.9900, Lack of fit, *p* > 0.05, 95% confidence level) using Equation (1). In this context, the experimental and predicted values of total soluble phenols were found to have a relationship, and in all cases, the error rates between them were smaller than 0.25% (Table 1). Similar trends in the error rates (from 0.05 to 2.53%) were reported previously during the optimization of UAE conditions for extracting phenolic compounds from muicle leaves using a Box–Behnken design and RSM analysis as statistical tools; moreover, the mathematical model showed good reliability between predicted and real data [27].

Total Soluble Phenols (mg/g DM) = 235.67 − 52.962X_ET_ + 8.735X_ET_^2^ − 4.637X_SA_ + 0.024X_SA_^2^ + 304.336X_PC_ − 126.340X_PC_^2^ + 1.027X_ET_ × X_SA_ − 0.149X_ET_^2^ × X_SA_ − 34.393X_ET_ × X_PC_ + 4.495X_ET_^2^ × X_PC_ − 0.938X_SA_ × X_PC_ (1)

where X_ET_: Extraction time (min); X_SA_: Sonication amplitude (%); X_PC_: pulse cycle (s).

**Figure 2:**
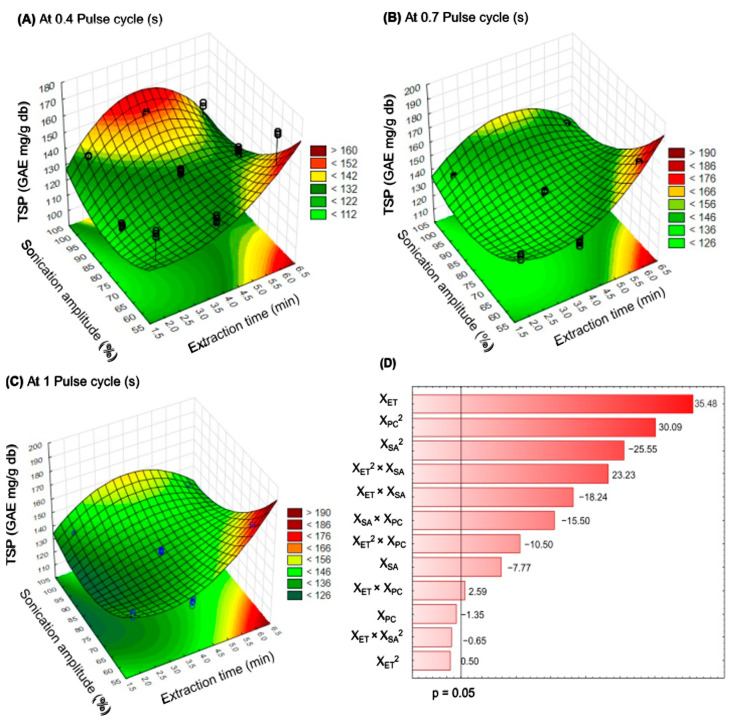
Response surface plots indicating the effect of Ultrasonic-Assisted Extraction on the total soluble phenols (TSP) content using at 0.4 (**A**), 0.7 (**B**), and 1.0 (**C**) of pulse cycle, and Pareto chart (**D**). X_ET_: extraction time, X_SA_: sonication amplitude, X_PC_: pulse cycle.

The significant interaction effects of UAE factors on total soluble phenols from PC leaves are shown in the three-dimensional response surface plots (Figure 2A–C), where their elliptical forms indicate the interactions between the evaluated factors (*p* < 0.05, R^2^ = 0.9925). In this study, the extraction of soluble phenols from PC leaves by ultrasound was observed under all the evaluated experimental conditions, independently from the extraction time (2, 4, and 6 min), sonication amplitude (60, 80, and 100%), and pulse cycle (0.4, 0.7, and 1 s). In the UAE, at 0.4 s or 1 s of the pulse cycle, an 80% sonication amplitude for 2 min typically results in the extraction of a low content of soluble phenols (Figure 2A,C) from PC leaves. Nevertheless, at a 0.7 s pulse cycle, the highest content of soluble phenols could be achieved using a 60% sonication amplitude for 6 min (Figure 2B). Additionally, Figure 2D shows the Pareto graph with the estimated effects (positive or negative) of the independent variables at a 95% confidence level, where extraction time (X_ET_) was the most important parameter when UAE was applied to extract soluble phenolic compounds from PC leaves. The optimization and control of the extraction time in the UAE process prevents phenolic degradation [27]. The principal effects (linear or quadratic) of independent variables and their interactions could be classified as X_ET_ > X_PC_ > X_SA_. Similar trends were reported by Aguilar-Hernández et al. [34] during the optimization of the UAE of phenolic compounds from soursop seeds (X_ET_ > X_PC_ > X_SA_); nonetheless, they mentioned that the estimated effects of independent variables are inherent for each plant matrix, and associated with their cellulose-hemicellulose-lignin ratio [35].

Additionally, the optimal UAE conditions for extracting soluble phenols from PC leaves are shown in Table 3. The best conditions were a 4 min extraction time, 100% sonication amplitude, and 0.6 s pulse cycle. The predicted response of total soluble phenols was 155.31 mg/g DM; moreover, it could range from 152.54 to 158.07 mg/g DM, with a 95% confidence level.

Only three studies were found related to the extraction of phenolic compounds by UAE from *Psidium cattleianum* by-products, including fruit peel [9] and leaves [12,18]. Maregalli et al. [9] evaluated the effect of indirect UAE (ultrasonic bath, 40 kHz, 154 W frequency) at 40 °C for 90 min on extracting anthocyanins from PC fruit peel. In PC leaves, Dacoreggio et al. [12] applied indirect UAE (ultrasonic bath, 70 W) for 180 min to extract phenolic compounds. Meanwhile, Coelho et al. [18] evaluated the effect of temperature (40, 50, and 60 °C), power ultrasound (100, 300, and 500 W), and the leaf-solvent ratio (1:10, 1:15, and 1:20) on polyphenol yield extraction using direct ultrasound coupled with a sonicator tip (20 kHz, 500 W, 5 min), with a complete factorial design 2^3^; however, no optimization of the UAE process was performed by the authors. To the best of our knowledge, this is the first study conducted to optimize the UAE conditions (pulse cycle, extraction time, and sonication amplitude) to obtain phenolic compounds from PC leaves, using a Box–Behnken design and response surface methodology. Moreover, these same independent variables were successfully investigated for optimizing the UAE process by RSM to extract bioactive compounds from soursop by-products (peel, seeds, pulp, and columella) [31,34], starfruit leaves [26], and muicle leaves [27].

### 2.3. Evaluation of Model Reliability and Comparison of Ultrasound-Assisted Extraction with Conventional Extraction

The optimal UAE conditions (extraction time = 4 min, pulse cycle = 0.6 s, sonication amplitude = 100%, Table 3) for phenolic compounds extraction from PC leaves were experimentally performed to verify the reliability of the mathematical model [33]. The experimental UAE of soluble phenolic compounds under the best conditions (158.18 mg/g DM, Table 4) was in agreement with the predicted values (from 152 to 158 mg/g DM, Table 3). Therefore, the optimized UAE process is a good technological alternative for polyphenol extraction from PC leaves, and similar tendencies have been reported by other researchers, who focused on the optimization of the UAE process to extract bioactive compounds from plant-based materials by RSM [26,27,31,34].

The soluble phenols and hydrolysable polyphenols, condensed tannins, the total polyphenolic content, and antioxidant activities (ABTS, DPPH, and FRAP) by UAE were compared with those obtained from a conventional aqueous–organic extraction method. The UAE effectiveness and phenolic yield were also compared (Table 4).

In general, significant differences (*p* < 0.05) were observed between UAE and the conventional aqueous–organic extraction (AOE) methods for all the evaluated variables; these findings comply with previous reports [26,27,31,34]. In PC leaf extracts, a higher concentration of soluble phenols (158.18 mg/g DM) and hydrolysable polyphenols (50.47 mg/g DM), condensed tannin (67.11 mg/g DM), and total phenolic compounds (275.75 mg/g DM) were observed when UAE was applied and compared to the AOE method (65.27, 40.45, 55.14, and 160.87 mg/g DM, respectively). Moreover, UAE exhibited a higher polyphenolic yield (27.75%) than the AOE (16.08%). Our results are in agreement with Aguilar-Hernández et al. [34], who compared UAE (extraction time of 5 min, sonication amplitude of 40%, and pulse cycle of 0.4 s) and conventional aqueous–organic extraction (methanol-acetone-water) for obtaining phenolic compounds from *Annona muricata* peel, where the UAE polyphenolic yield was >50% higher than conventional aqueous–organic extraction, demonstrating that UAE exhibited higher extraction yields in a shorter extraction time. Similar trends were reported by Anaya-Esparza et al. [27] when comparing UAE (10.82%) with stirring (9.92%) and thermal decoction (9.55%) in muicle leaves. These results are attributed to the physical and chemical effects of ultrasound cavitation that disrupt the cell wall, facilitating the release of phenolic compounds [36]. Additionally, the UAE was more effective (1.71-times) in extracting polyphenols than the AOE, reducing the extraction time by 96.66%.

To establish the overall value of the optimized UAE extraction process to obtain phenolic compounds from PC leaves, the data were compared with other reported studies (Table 5). Here, the total soluble phenols content and yields obtained in this study by UAE are among the highest extraction yields (158.18 mg/g DM) obtained within four minutes of operation. These comparisons include other extraction techniques such as aqueous–organic (65.27 mg/g, 120 min), ultrasound coupled with sonicator tip (25.5 mg/g, 5 min) [18], ultrasound bath (101 mg/g, 180 min), enzymatic techniques using a cellulose complex (121 mg/g, 360 min) [12], supercritical fluid using CO_2_ (0.363 mg/g, 180 min), Soxhlet (0.49 mg/g, 360 min), hydrodistillation (0.252 mg/g, 180 min) [1], pressurized liquid (4.43 mg/g, 20 min), aqueous infusion (0.067 mg/g, 10 min) [4], maceration with ethanol (3.197 mg/g, 120 min) [37], and stirring with methanol (157.2 mg/g, 4320 min) [22]. Likewise, it is also evident that this comparison requires other factors that may influence the polyphenolic content of PC leaves, such as the season of collection, leaf color, maturity stage, and the drying and storage conditions [12,36].

Diverse studies have demonstrated that PC leaf extracts exhibit antioxidant properties using different in vitro tests, including DPPH, ABTS, FRAP, and alkyl and hydroxyl radical scavenging activities [12,18,22,38,39]. Table 6 lists the values of the antioxidant capacities of the PC leaf extracts obtained by UAE and the AOE. Significant (*p* < 0.05) differences were observed between extraction methods, where the PC leaf extracts by UAE exhibited the highest antioxidant capacities compared to the AOE method in all of the evaluated in vitro tests (ABTS, DPPH, and FRAP). Conversely, it has been reported that PC leaf extracts obtained by UAE (using an ultrasound bath) exhibited lower antioxidant properties using the DPPH test than those obtained with the enzymatic method [12]. Moreover, muicle leaf extracts obtained using UAE showed lower antioxidant capacities (ABTS, DPPH, and FRAP) than the thermal decoction extracts [27]. These differences could be associated with each plant matrix and the extracted phenolic profile (chemical structure and the position and number of hydroxyl groups), which significantly influence the antioxidant capacity [26]. Moon et al. [22] reported that the acetate fraction from PC leaves exhibited the highest antioxidant capacity by DPPH than butanol, methanol, water, hexane, and chloroform fractions, associated with the content and kind of polyphenolic compounds in each fraction. Furthermore, the order of antioxidant capacity from the PC leaf extract for both evaluated extraction methods was DPPH > FRAP > ABTS. These results may be associated with the extracted phenols and their mechanism to neutralize radicals such as chelating metals, donating electrons, or transferring electrons [38].

### 2.4. Soluble Polyphenols Profile

**Figure 3:**
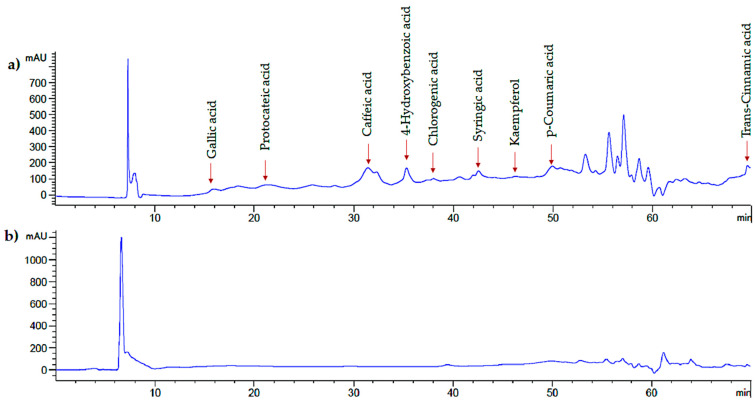
Soluble polyphenols chromatographic profile of *Psidium cattleianum* leaf extract by ultrasound-assisted extraction (**a**) and aqueous–organic extraction (**b**).

The identification of phenolic compounds from PC leaf extracts obtained by optimal UAE conditions (X_ET_ = 4 min, X_PC_ = 0.6 s, and X_SA_ = 100%) and conventional aqueous–organic extraction was performed with HPLC (Figure 3).

Nine phenolic compounds were identified in the UAE leaf extracts, including phenolic acids and flavonoids such as gallic, protocateic, chlorogenic, caffeic, p-coumaric, trans-cinnamic, 4-hydroxybenzoic, and syringic acids, and kaempferol. Furthermore, with the conventional aqueous–organic extraction the same phenolic compounds were identified, except for gallic and p-coumaric acids that were not detected (Table 7). In general, UAE exhibited a higher phenolic content (*p* < 0.05) than the conventional extraction aqueous–extraction method. In UAE, the highest content of phenolic compounds was found for kaempferol (727 mg/100 g DM), 4-hydroxybenzoic acid (713.76 mg/100 g DM), and protocateic acid (285.02 mg/100 g DM). In contrast, in the conventional aqueous–organic extraction method, kaempferol (549.02 mg/100 g DM), 4-hydroxybenzoic acid (124.62 mg/100 g DM), and chlorogenic acid (99.12 mg/100 g DM) had the highest content of phenolic compounds. Conventional techniques employed to extract bioactive compounds from plant materials are characterized by poor quality because they can cause degradation [34]. In turn, UAE is a technological and green alternative for extracting phenolic compounds from PC leaves.

Studies regarding the phytochemical profile characterization of PC leaf extracts are scarce [4,22,40]. Ferulic acid was qualitatively identified from a chloroform fraction [40]; and gallic acid, quercetin, guaijaverin, and naringing were qualitatively identified from an ethanolic fraction [22], using the stirring extraction method (3 days at room temperature). Zandoná et al. [4] characterized the phytochemical profile of PC leaf extracts obtained from an aqueous infusion, pressurized liquid extraction with water (PLEW), PLEW + ethanol (PLEWE), and pressurized liquid extraction with ethanol (PLEE). They found differences between the extracted phenolics (type and contents) and extraction methods. With the PLEE and PLEWE methods, the highest phenolic compounds were catechin (1732 and 1388 µg/g, respectively), vanillic acid (1526 and 1492 µg/g, respectively), and gallic acid (105 and 111 µg/g, respectively), and 4-hydroxybenzoic, chlorogenic, caffeic, syringic, and ellagic acids, myricetin, luteolin, quercetin, and kaempferol were also identified. Furthermore, in PLEW, catechin (1999 µg/g), vanillic acid (2326 µg/g), and gallic acid (32.11 µg/g) exhibited the highest content, and the previously mentioned compounds were also identified, except for syringic acid and myricetin. However, caffeic and syringic acids, myricetin, luteolin, and quercetin were not detected in aqueous infusion extracts. In contrast, the other compounds were detected in small quantities (vanillic acid = 28.20 µg/g, gallic acid = 1.20 µg/g, and catechin = 37.26 µg/g) compared to the other extraction methods, demonstrating the influence of extraction methods on the phytochemical profile of PC leaf extracts. No information was found on the screening of the phytochemical profile of PC leaf extracts obtained by the UAE.

## 3. Materials and Methods

The experimental work was carried out in two phases. The first phase was conducted to obtain the highest soluble phenolic compounds from *P. cattleianum* leaves under UAE using the response surface methodology. The second phase consisted of validating UAE optimal conditions experimentally and comparing them with a conventional aqueous–organic extraction procedure.

### 3.1. Plant Material and Chemicals

*Psidium cattleianum* leaves (PC) of a light green color were collected from wild trees in Tepic, Nayarit, Mexico, in May 2021. PC leaves were washed with distilled water and oven-dried (Memmert GmbH, Schwabach, Germany) at 50 °C for 24 h. Then, leaves were grounded in a food processor (Nutribullet NB-101B, Los Angeles, CA, USA) and sieved using a stainless-steel mesh (no. 35; Fisher Scientific, Hampton, NH, USA) to a particle size of 500 µm (Figure 1). The PC leaf powder was stored at 25 °C and protected from light prior to analysis.

All chemicals and reagents were of analytical or HPLC grade. Phenolic standards, Folin–Ciocalteu phenol reagent, 1,1-Diphenyl-2-picrylhydrazyl, 2,2_-Azinobis-(3-ethylbenzothiazoline-6-sulfonic acid), 2,4,6-tripyridyl-s-triazine, 6-hydroxy-2,5,7,8-tetramethylchroman-2-carboxylic acid, ferric chloride hexahydrate, sodium acetate, trifluoroacetic acid, water, acetonitrile, acetic acid, and methanol were obtained from Sigma-Aldrich Co. (St. Louis, MO, USA).

### 3.2. First Phase

#### 3.2.1. Experimental Design

Considering a randomized experiment, a three-level, three factor Box–Behnken design was applied to obtain the optimal conditions for extracting total soluble phenols from PC leaves using UAE, including sonication amplitude (X_SA_, 60, 80, and 100%), pulse cycle (X_PC_, 0.4, 0.7, and 1.0 s), and extraction time (X_ET_, 2, 4, and 6 min). The experimental design used in this study consisted of 15 different combinations of factors and their levels (Table 1).

#### 3.2.2. Ultrasound-Assisted Extraction (UAE) Procedure of Polyphenolic Compounds

UAE was adapted according to the methodology proposed by Coehlo et al. [18] with some modifications. For the extraction of phenols, a high-intensity ultrasonic processor of 400 Watts of nominal output power and 24 kHz frequency (UP400S, Hielscher Ultrasonics, Teltow, Germany) coupled with an ultrasonic probe (H7, Tip 7, 300 W/cm^2^ of acoustic density power, Hilscher, Teltow, Germany) was used. The dried PC leaf (500 mg) powder was mixed with 50 mL of an acetone: water solution (80:20 *v*/*v*) and sonicated (the ultrasonic probe was immersed 2 cm into the extraction solution) following the experimental design conditions (Table 1). The UAE was performed using a cold-water bath to maintain the extraction temperature in a range of 20 ± 1 °C. After UAE, the extracts were centrifuged (at 8000× *g* for 10 min at 4 °C, Hermle Z32HK, Wehingen, Germany) and supernatants were recovered; they were also stored at −20 °C until analysis [34].

#### 3.2.3. Total Soluble Phenols

From the recovered supernatants, the total soluble phenols content was quantified according to Montreau [40] with a slight modification. In a 96-well microplate, the PC leaf extract (12 µL) was mixed with the Folin–Ciocalteu reagent (12 µL), sodium bicarbonate (116 µL of a solution at 75 g/L), and distilled water (164 µL), and was then homogenized in darkness for 15 min. Lastly, the absorbance was measured at 750 nm using a microplate reader (800TS, Biotek, Winooski, VT, USA) and the results were expressed as milligrams of gallic acid equivalents per gram of dry weight (mg/g DM), calculated using a gallic acid standard curve (R^2^ = 0.991).

#### 3.2.4. Ultrasound-Assisted Extraction (UAE) Yield of Polyphenolic Compounds

The UAE yield was expressed as a percentage and was calculated using Equation (2) [33].

(2) $$\mathrm{Yield}\;(\%)=\frac{\mathrm{Total}\;\mathrm{soluble}\;\mathrm{phenols}\;(g)}{\mathrm{Dried}\;\mathrm{leaf}\;\mathrm{powder}\;(g)}$$

#### 3.2.5. Response Surface Methodology Analysis

Once the experimental phenolic compound extraction using UAE was performed, the response surface methodology was applied to obtain the optimal UAE conditions for extracting soluble phenols from PC leaves. A second-order polynomial (Equation (3)), which includes all terms) was used to calculate the predicted response:

(3) $$Y=\beta 0+\overset{Ε}{\underset{i=A}{\sum }}\beta i\;\mathrm{Xi}+\overset{Ε}{\underset{i=A}{\sum }}\overset{Ε}{\underset{j=A\neq i}{\sum }}\beta \mathrm{ij}\;\mathrm{Xi}+Ε$$

where Y is the predicted response (total soluble phenols), X_i_ represent values for the studied factors (X_SA,_ X_PC_, and X_SA_), β_0_ is a constant, β_i_ is the main effect coefficient for each variable and β*_ij_* are the interaction effect coefficients. Model adequacy was evaluated using an F ratio. The lack of fit test (*p* > 0.05) and coefficient of determination (R-square and R-Adjust) were used to evaluate the adequacy and precision of the mathematical model [41]. Results of the experimental design were fitted with a second-order polynomial equation using a multiple regression technique. Statistical analyses were performed using Statistical software (Statistic v. 12.5 Statsoft^®^. Tulsa, OK, USA).

### 3.3. Second Phase

#### 3.3.1. Model Reliability and Comparison of Ultrasound-Assisted Extraction with Conventional Aqueous–Organic Extraction

The optimized UAE parameters obtained from the RSM analysis were experimentally verified to evaluate the model accuracy. These results were compared with the total soluble phenols content obtained by conventional aqueous–organic extraction, and the effectiveness of UAE was calculated using Equation (4) [31]. Hydrolysable polyphenols, condensed tannin, antioxidant activity, and the phenolic profile were also compared.

(4) $$\mathrm{Effectiveness}\;(n-\mathrm{times})=\frac{\mathrm{Total}\;\mathrm{polyphenols}\;\mathrm{by}\;\mathrm{UAE}\;(g)}{\mathrm{Total}\;\mathrm{polyphenols}\;\mathrm{by}\;\mathrm{conventional}\;\mathrm{extraction}\;(g)}$$

#### 3.3.2. Conventional Aqueous–Organic Extraction of Polyphenols

The conventional extraction of total soluble phenols was performed following the method of Pérez-Jiménez et al. [42]. The dried PC leaf (500 mg) powder was mixed with 25 mL of the methanol–water solution (50:50, *v/v*) acidified with HCl (0.8% *v/v* 16 mM HCl) and stirred at room temperature (25 ± 1 °C) in a shaker (Heidolph Rex 2, Heidoplh Instruments, Schuwabach, Germany) for 60 min at a moderate speed (10× *g*). Then, the extracts were centrifuged for 10 min (at 8000× *g* at 4 °C), and supernatants were recovered; the residues were resuspended in 25 mL of the acetone: water solution (80:20 *v/v*) and treated under the same conditions of stirring and centrifugation as described earlier. Both supernatants were combined to measure soluble phenols, while residues were employed to quantify hydrolysable phenols and condensed tannins.

#### 3.3.3. Total Soluble Phenols, Hydrolysable Polyphenols, and Condensed Tannins

Total soluble phenols were quantified as described in Section 3.2.3. The hydrolysable polyphenols were determined according to Hartzfeld et al. [43]. For this, 500 mg of dried residues were obtained after the residues were hydrolyzed with methanol/H_2_SO_4_ (90/10 *v/v*, 20 h at 85 °C). Samples were then centrifuged at 5000× *g* for 10 min at 25 °C, and supernatants were recovered; later, the residues were resuspended twice with distilled water (10 mL) and then centrifuged. All supernatants were combined, hydrolysable polyphenols were determined in the supernatants using the Folin–Ciocalteu reagent (see Section 3.2.3), and the results were expressed as mg/g DM. Regarding condensed tannins [44], 500 mg of dried residues were hydrolyzed with butanol/HCl/FeCl3 (10 mL, 97.5:2.5 *v/v*) solution at 100 °C for 3 h; afterward, samples were centrifuged (6000× *g* for 10 min at 4 °C). Condensed tannins were spectrophotometrically (800TS, Biotek, Winooski, VT, USA) quantified at 550 nm in the supernatants, and the results were expressed as milligrams per gram dry matter (mg/g DM), which were calculated using the Mediterranean carob pod (*Ceratonia siliqua* L.) standard curve (R^2^ = 0.993).

#### 3.3.4. Antioxidant Capacity

From the UAE and conventional aqueous–organic extracts, the antioxidant capacity was determined with three different in vitro assays, including ABTS (2,2′-Azinobis-(3-ethylbenzothiazoline-6-sulfonic acid) radical cation scavenging and DPPH (1,1-Diphenyl-2-picrylhydrazyl) radical scavenging activities, as well as FRAP (ferric-ion reducing antioxidant power).

Regarding the ABTS assay, the leaf extract (35 µL) was mixed with ABTS radical (265 µL at 7 mM); then, the reaction mixture was shaken in darkness for 7 min, and the absorbance was measured at 734 nm [45]. For the DPPH assay, the leaf extract (40 µL) reacted with the DPPH solution (190 µL at 190 mM), and after 10 min of stirring in the dark, absorbance was measured at 734 nm [46]. In relation to the FRAP assay, 264 µL of the FRAP solution (10:1:1 *v/v/v*) composed of sodium acetate buffer (0.3 M, pH 3.6), ferric chloride hexahydrate (20 mM), and 2,4,6-tripyridil-s-triazine (10 mM) was mixed with the leaf extract (36 µL) and distilled water (9 µL), and after 30 min of stirring in the dark, the absorbance was measured at 595 nm [47].

A 96-well microplate reader (800TS, Biotek, Winooski VT, USA) was used to measure the absorbance of all antioxidant capacity methods. The results were expressed as mmol Trolox (6-hydroxy-2,5,7,8-tetramethylchroman-2-carboxylic acid) equivalent per gram (mmol/g DM), which were calculated using a Trolox standard curve (R^2^ = 0.996, 0.996, 0.998, respectively).

#### 3.3.5. Identification of Phenolic Compounds from PC Leaf Extracts by HPLC

The identification of the phenolic compounds of PC leaf extracts obtained under optimal UAE conditions and conventional aqueous–organic extraction was performed following the methodology developed by Aguilar-Hernández et al. [34], with some modifications. Briefly, the extracts were concentrated to dryness, subsequently resuspended in 1 mL of acidified water with 2% acetic acid and filtered through 0.22 µm membrane filters. Samples (30 μL) were injected into an HPLC system (Agilent Technologies 1260 Infinity, Waldbronn, Germany) equipped with a photodiode array detector and a C18 reverse-phase column (250 mm × 4.6 mm, 5 μm; Thermo Scientific, Sunnyvale, CA, USA). The eluents were acidified water with 2% acetic acid (eluent A) and acidified water (0.5% acetic acid)-methanol (10:90 *v/v*, eluent B). Standards and samples were analyzed using a gradient as follows: 0% B; 0–35 min, 35% B; 35–55 min, 75% B; 55–60 min, 100% B; 60–70 min; 0% B, at a flow rate of 0.4 mL/min. Peak areas in the samples were detected from 280 to 320 nm and calibration curves for gallic, protocatechuic, chlorogenic, caffeic, neochlorogenic, caffeic, p-coumaric, trans-cinnamic, 4-hydroxybenzoic, and syringic acids, and kaempferol were used to identify and quantify the peaks.

### 3.4. Statistical Analysis

Data were expressed as mean ± standard deviation. In the first phase, data were examined using response surface methodology, and the Tukey test was used to examine the differences between samples (*p* < 0.05). In the second stage, the Student *T* test was applied (*p* < 0.05) to examine the differences between UAE and the conventional aqueous–organic extraction method. All statistical analyses were performed using Statistical software (Statistic v. 12.5 Statsoft^®^, Tulsa, OK, USA).

## 4. Conclusions

In this study, the optimization of the extraction of phenolic compounds from *Psidium cattleianum* (PC) leaves by ultrasound-assisted extraction (UAE), employing pulse cycle, sonication amplitude, and extraction time, was performed using the response surface methodology (RSM). In the UAE, a 60% sonication amplitude at 0.6 s of the pulse cycle resulted in the maximum extraction yield of total phenols within the four min of operation. The UAE was more effective (1.71-times) for extracting polyphenolic compounds with a strong antioxidant capacity than the conventional aqueous–organic method, reducing the extraction time by 96.66%. The UAE leaf extracts identified a number of phenolic compounds including gallic, protocateic, chlorogenic, caffeic, p-coumaric, trans-cinnamic, 4-hydroxybenzoic, and syringic acids, and kaempferol. The content of these phenolic compounds was higher for UAE than for conventional aqueous–organic extraction. The present study highlights the use of the UAE as a green technique to improve the yield extraction of the PC leaf phytochemicals with antioxidant capacity. Moreover, PC leaf extracts are widely used for therapeutic and industrial purposes; thus, they can be potentially used as a functional ingredient for developing food (for humans or animals) and nutraceuticals with beneficial human health effects. On the other hand, further studies are needed to complete the phytochemical characterization of PC leaf extracts to validate their potential uses.

## Figures and Tables

**Table 1:** Experimental matrix used for response surface methodology with experimental and predicted values for the independent variables, error rate, yield, and final temperature after ultrasound-assisted extraction of *Psidium cattleianum* leaf extracts.

Run	Predictors ^1^			Response Variables		Error Rate (%)	Yield (%)	Final Temperature (°C)
	X_PC_ (s)	X_SA_ (%)	X_ET_ (min)	Experimental TSP ^2^	Predicted TSP ^3^			
1	0.7	60	2	136.88 ± 2.11 de	137.05	−0.12	13.68	20 ± 1.0
2	0.7	60	6	159.55 ± 0.84 a	169.38	−0.10	15.95	21 ± 0.5
3	0.7	100	2	137.96 ± 0.48 d	138.13	−0.12	13.79	20 ± 0.5
4	0.7	100	6	144.14 ± 1.24 c	143.97	0.11	14.41	20 ± 0.5
5	0.4	80	2	116.24 ± 0.53 h	116.07	0.14	11.62	19 ± 0.5
6	0.4	80	6	133.11 ± 1.31 ef	133.28	−0.12	13.31	20 ± 0.5
7	1	80	2	117.26 ± 1.51 h	117.05	0.17	11.72	20 ± 1.0
8	1	80	6	137.86 ± 1.48 d	138.02	0.11	13.78	21 ± 0.5
9	0.4	60	4	128.44 ± 1.60 g	128.44	0	12.84	20 ± 0.5
10	0.4	100	4	151.39 ± 0.68 b	151.39	0	15.13	20 ± 0.5
11	1	60	4	131.77 ± 1.66 fg	131.77	0	13.17	20 ± 0.5
12	1	100	4	132.21 ± 1.51 fg	132.21	0	13.21	20 ± 0.5
13	0.7	80	4	137.73 ± 0.58 d	137.67	0.07	13.77	21 ± 0.5
14	0.7	80	4	137.98 ± 0.90 d	137.67	0.22	13.79	21 ± 0.5
15	0.7	80	4	137.70 ± 0.87 d	137.67	0.02	13.77	21 ± 0.5

All values are mean ± standard deviation of three determinations by triplicate (*n* = 9). Different letters in each file indicate significant statistical differences between treatments (α= 0.05). ^1^ Pulses cycle (X_PC_); Sonication Amplitude (X_SA_) and Extraction Time (X_ET_); ^2^ Total soluble phenols (TSP, mg/g dry matter); ^3^ The values were predicted using a secondary polynomial equation, R^2^ = 0.99.

**Table 2:** Analysis of variance using a quadratic model, with the ultrasound-assisted extraction conditions on the total soluble phenols content from *Psidium cattleianum* leaf extracts.

Source ^1^	Analysis of Variance				Regression Coefficients
	SS ^2^	DF ^3^	MS ^4^	F Value	Total Soluble Phenolics β-Coefficient
Mean/intercept	-	-	-	-	235.671 *
X_ET_	2185.80	1	2185.80	1382.63 *	−52.962 *
X_ET_^2^	0.406	1	0.406	0.257 **	8.735 *
X_SA_	95.66	1	95.66	60.51 *	−4.637 *
X_SA_^2^	1032.46	1	1032.46	653.08 *	0.024 *
X_PC_	2.913	1	2.913	1.843 **	304.336 *
X_PC_^2^	1432.13	1	1432.13	905.89 *	−126.340 *
X_ET_ * X_SA_	526.02	1	526.02	332.73 *	1.027 *
X_ET_^2^ * X_SA_	853.83	1	853.83	540.092 *	−0.149 *
X_ET_ * X_PC_	10.62	1	10.62	6.719 *	−34.393 *
X_ET_^2^ * X_PC_	174.58	1	174.58	110.43 *	4.495 *
X_SA_ * X_PC_	379.91	1	379.91	240.13 *	−0.938 *
Lack of fit	0.673	1	0.673	0.426 **	
Pure error	50.58	32	1.58		
R-square	0.9925				
R-Adjust	0.9900				
Total SS	6888.34				

^1^ Extraction time (X_ET_), Sonication amplitude (X_SA_), and Pulse cycle (X_PC_). ^2^ SS, sum of square. ^3^ DF, degree of freedom. ^4^ MS, means square. * Significant (*p* < 0.05), ** non-significant (*p* > 0.05).

**Table 3:** Optimal conditions for ultrasonic-assisted extraction for the content of total soluble phenols from *Psidium cattleianum* leaf extracts obtained using the predicted model.

Parameter	Total Soluble Phenols (mg/g DM)
Extraction time (min)	4
Pulse cycle (s)	0.6
Sonication amplitude (%)	100
Optimal response	155.31
−95% Confidence Limit	152.54
+95% Confidence Limit	158.07

**Table 4:** Total soluble phenols, hydrolysable polyphenols, condensed tannins, and total polyphenols from *Psidium cattleianum* leaf extracts using the optimal conditions for ultrasound-assisted extraction and conventional aqueous–organic extraction, yield, and effectiveness.

Parameter	UAE	AOE
Total Soluble Phenols (mg/g DM)	158.18 ± 2.00 a	65.27 ± 3.85 b
Hydrolysable polyphenols (mg/g DM)	50.47 ± 2.98 a	40.45 ± 2.08 b
Condensed tannins (mg/g DM)	67.11 ± 2.18 a	55.14 ± 3.23 b
Total polyphenols (mg/g DM)	275.75 ± 2.39 a	160.87 ± 3.06 b
Polyphenolic yield (%)	27.75	16.08
Effectiveness UAE (n-times)	1.71	

All values are mean ± standard deviation of three determinations (*n* = 9). Different letters in each file indicate significant statistical differences between treatments (*p* < 0.05). Experimental conditions for ultrasound-assisted extraction (UAE): X_ET_ = 4 min, X_PC_ = 0.6 s, and X_SA_ = 100%. Experimental conditions for aqueous–organic extraction (AOE)= 2 h under shaking.

**Table 5:** Review of the total soluble phenols content from *Psidium cattleianum* leaves reported according to different extraction methods.

Extraction Method	Solvent	Extraction Time (min)	Temperature (°C)	^2^ TSP (mg/g DM)	^3^ Yield (%)	Ref.
Ultrasound (sonicator tip)	Acetone-water	4	20	158.18	15.81	This work
shaking	Methanol-acetone-water	120	25	65.27	6.52	This work
Ultrasound (sonicator tip)	Hexane	5	60	25.5	2.55	[18]
Ultrasound bath	Water	180	NI	101	10.1	[12]
Enzymatic	Water	360	45	121	12.1	[12]
Supercritical fluid	CO_2_	180	50 °C	0.363	0.03	[1]
Soxhlet	Petroleum ether	360	Boiling	NI	0.49	[1]
Hydro-distillation	Water	180	100	NI	0.40	[1]
Pressurized liquid	Water	20	50	4.43	0.44	[4]
Aqueous infusion	Water	10	80	0.067	<0.01	[4]
Maceration	Ethanol-water	120	RT	3.197	0.31	[37]
^1^ Stirring	Methanol	4320	RT	157.2	15.72	[22]

^1^ Extract was fractioned and concentrated; NI: no information; RT: at room temperature. ^2^ Total soluble phenols (TSP) in milligram of gallic acid equivalents per gram of dry weight (mg/g DM), ^3^ Yield was calculated using Equation (2).

**Table 6:** Antioxidant capacity of *Psidium cattleianum* leaf extracts by optimal UAE conditions and conventional aqueous–organic extraction.

Antioxidant Activity	UAE	AOE
ABTS (mmol/g DM)	237.38 ± 4.49 a	211.05 ± 5.11 b
DPPH (mmol/g DM)	418.19 ± 4.32 a	282.83 ± 3.67 b
FRAP (mmol/g DM)	405.19 ± 3.61 a	262.75 ± 5.39 b

All values are mean ± standard deviation of three determinations (*n* = 9). Different letters in each file indicate significant statistical differences between treatments (*p* < 0.05). DM: Dry matter. Experimental conditions for ultrasound-assisted extraction (UAE): X_ET_ = 4 min, X_PC_ = 0.6 s, and X_SA_ = 100%. Experimental conditions for aqueous–organic extraction (AOE) = 2 h under shaking.

**Table 7:** Phenolic compounds profile from *Psidium cattleianum* leaf extracts using the optimal conditions of ultrasound-assisted extraction and conventional aqueous–organic extraction.

No.	Compound	Retention Time (min)	UAE ^1^ (mg/100 g DM)	AOE ^2^ (mg/100 g DM)
1	Gallic acid	15.957	42.20 ± 0.79 a	nd
2	Protocateic acid	21.376	285.02 ± 0.85 a	68.15 ± 6.60 b
3	Chlorogenic acid	35.305	106.82 ± 1.16 a	99.12 ± 0.83 b
4	Caffeic acid	38.030	27.83 ± 1.30 a	17.33 ± 0.36 b
5	p-Coumaric acid	49.948	63.911 ± 1.5 a	nd
6	Trans-Cinnamic acid	69.641	17.00 ± 0.01 a	13.60 ± 0.06 b
7	4-Hydroxybenzoic acid	31.409	713.76 ± 30.37 a	124.62 ± 6.71 b
8	Syringic acid	42.549	185.77 ± 3.61 a	55.05 ± 0.85 b
9	Kaempferol	46.208	727.22 ± 21.89 a	549.62 ± 17.56 b

All values are mean ± SD of three determinations. nd: not detected. Different letters in each file indicate significant statistical differences between treatments (*p* < 0.05). ^1^ Experimental conditions for ultrasound-assisted extraction (UAE): X_ET_ = 4 min, X_PC_ = 0.6 s, and X_SA_ = 100%. ^2^ Experimental conditions for aqueous–organic extraction (AOE) = 2 h under stirring.

## Data Availability

The dataset used and/or analyzed during the current study are available from the corresponding author on reasonable request.

## Sample Availability

Samples of the compounds are not available from the authors.
